# Bis(tetra­phenyl­phospho­nium) bis­[*N*-(phenyl­sulfon­yl)dithio­carbimato-κ^2^ *S*,*S*′]platinate(II) monohydrate

**DOI:** 10.1107/S1600536810027364

**Published:** 2010-07-21

**Authors:** H. A. Silvério, S. Guilardi, Wilson P. Flauzino Neto, Raquel S. Amin, Marcelo R. L. Oliveira

**Affiliations:** a Instituto de Química – UFU, 38408-100 Uberlândia, MG, Brazil; b Departamento de Química – UFV, 36571-000 Viçosa, MG, Brazil

## Abstract

The asymmetric unit of the title compound, (C_24_H_20_P)_2_[Pt(C_7_H_5_NO_2_S_3_)_2_]·H_2_O, consists of two tetra­phenyl­phospho­nium cations, two half bis­[*N*-(phenyl­sulfon­yl)dithio­carbim­ato]platinate(II) dianions and one water mol­ecule. The anions are completed by crystallographic inversion symmetry associated with the central Pt^II^ ion. The Pt^II^ ion is doubly *S*,*S*′-chelated by two symmetry-related phenyl­sulfonyl­dithio­carbimate ligands, forming a slightly distorted square-planar configuration. Besides the electrostatic attraction between oppositely charged ions in the crystal packing, intra­molecular C—H⋯O and several inter­molecular C—H⋯O, C—H⋯N and O—H⋯O hydrogen-bonding inter­actions between the cations, anions and water mol­ecules are observed.

## Related literature

For general background to Pt complexes, see: Faraglia *et al.* (2001 ▶). Dithio­carbimatoplatinate(II) complexes with tetra­butyl­ammonium counter cations were reported by Amim *et al.* (2008 ▶); Oliveira *et al.* (2004 ▶) and with tetra­phenyl­phospho­nium by Guilardi *et al.* (2010 ▶). For the structures of related dithio­carbimates, see: Oliveira *et al.* (2003 ▶); Franca *et al.* (2006 ▶). For reference structural data, see: Allen *et al.* (1987 ▶).
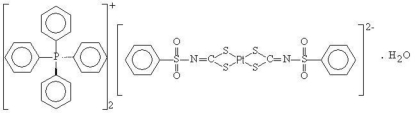


## Experimental

### 

#### Crystal data

(C_24_H_20_P)_2_[Pt(C_7_H_5_NO_2_S_3_)_2_]·H_2_O
*M* *_r_* = 1354.45
Triclinic, 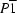

*a* = 9.2972 (1) Å
*b* = 13.6482 (3) Å
*c* = 24.4279 (5) Å
α = 105.440 (1)°
β = 90.916 (1)°
γ = 107.777 (1)°
*V* = 2829.11 (9) Å^3^
*Z* = 2
Mo *K*α radiation
μ = 2.81 mm^−1^
*T* = 120 K
0.62 × 0.23 × 0.16 mm

#### Data collection

Nonius KappaCCD diffractometer
Absorption correction: Gaussian (Becker & Coppens, 1974 ▶) *T*_min_ = 0.275, *T*_max_ = 0.662
20391 measured reflections
10825 independent reflections
9007 reflections with *I* > 2σ(*I*)
*R*_int_ = 0.025

#### Refinement

*R*[*F* ^2^ > 2σ(*F* ^2^)] = 0.026
*wR*(*F* ^2^) = 0.063
*S* = 1.03
10825 reflections
706 parameters
H-atom parameters constrained
Δρ_max_ = 0.53 e Å^−3^
Δρ_min_ = −1.41 e Å^−3^

### 

Data collection: *COLLECT* (Nonius, 2000 ▶); cell refinement: *DENZO* and *SCALEPACK* (Otwinowski & Minor, 1997 ▶); data reduction: *DENZO* and *SCALEPACK*; program(s) used to solve structure: *SHELXS86* (Sheldrick, 2008 ▶); program(s) used to refine structure: *SHELXL97* (Sheldrick, 2008 ▶); molecular graphics: *ORTEP-3 for Windows* (Farrugia, 1997 ▶); software used to prepare material for publication: *WinGX* (Farrugia, 1999 ▶).

## Supplementary Material

Crystal structure: contains datablocks global, I. DOI: 10.1107/S1600536810027364/wm2374sup1.cif

Structure factors: contains datablocks I. DOI: 10.1107/S1600536810027364/wm2374Isup2.hkl

Additional supplementary materials:  crystallographic information; 3D view; checkCIF report

## Figures and Tables

**Table:** 

S11—Pt1	2.3111 (7)
S12—Pt1	2.3171 (7)
Pt2—S21	2.3130 (8)
Pt2—S22	2.3299 (7)

**Table:** 

S11—Pt1—S12	74.95 (3)
S21—Pt2—S22	74.22 (3)

**Table 2:** Hydrogen-bond geometry (Å, °)

*D*—H⋯*A*	*D*—H	H⋯*A*	*D*⋯*A*	*D*—H⋯*A*
O*W*—H1*W*⋯O12^i^	0.85	2.01	2.845 (3)	166
O*W*—H2*W*⋯O22^ii^	0.85	1.99	2.831 (4)	172
C16—H16⋯O21^ii^	0.95	2.39	3.230 (4)	148
C17—H17⋯O11	0.95	2.5	2.889 (4)	105
C24—H24⋯O11^iii^	0.95	2.39	3.154 (4)	137
C25—H25⋯O*W*	0.95	2.46	3.303 (4)	149
C27—H27⋯O21	0.95	2.56	2.926 (4)	103
C110—H110⋯N11^iv^	0.95	2.58	3.371 (4)	141
C112—H112⋯O11^v^	0.95	2.51	3.292 (4)	139
C123—H123⋯N11^iii^	0.95	2.61	3.385 (4)	139

Symmetry codes: (i) 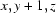; (ii) 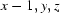; (iii) 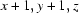; (iv) 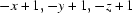; (v) 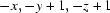.

**Fig. 1.**
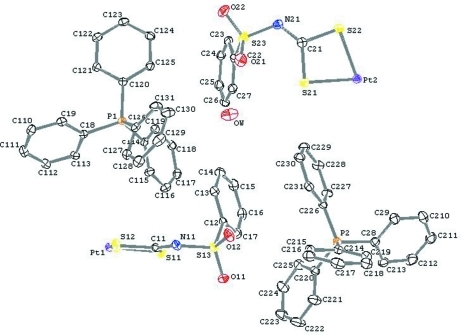
ORTEP view of the asymmetric unit of (I), showing the atom labelling. Displacement ellipsoids are drawn at the 30% probability level. H atoms have been omitted for clarity.

**Fig. 2.**
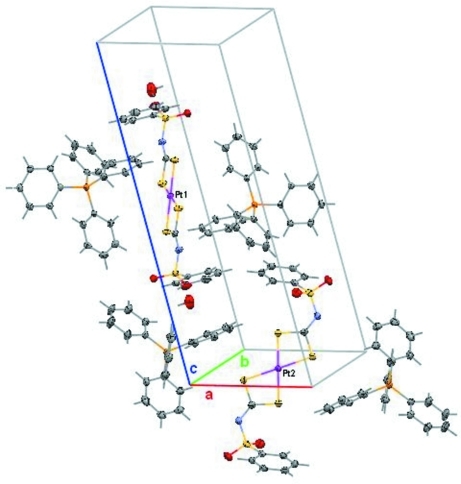
Part of the crystal packing of the structure of (I).

## supplementary crystallographic information

### Comment

The interest in platinum(II) complexes containing S donors has increased in recent years, with the aim of synthesizing anti-tumor drugs having reduced toxicity with respect to *cis*-platin (Faraglia *et al.*, 2001). The structures of anionic platinum-dithiocarbimato complexes with general formula [Pt(*R*SO_2_N═CS_2_)]^2-^ (*R* = aryl groups) have been determined by X-ray diffraction techniques. Some of them have the tetrabutylammonium cation as counter ion (Amim *et al.*, 2008; Oliveira *et al.*, 2004), but only one has the tetraphenylphosphonium as counter ion (Guilardi *et al.*, 2010). Variations in the counter ions and in the *R* groups may be important to modulate the activity of these compounds favoring the biological application.

The two Pt(II) atoms in the title compound are located at inversion centres. In the unit cell there are two anions, four cations and two water molecules (Figs. 1, 2). The Pt(II) atoms are coordinated by the four sulfur atoms of the two *N*-(phenylsulfonyldithiocarbimato) ligands within a distorted square-planar configuration (the bite angles S1—Pt—S2 are smaller than 90°). The four Pt—S bond lengths are almost equal (Table 1). In the fragment N═CS_2_, the C—S bond lengths are nearly equal and are shorter than typical C—S single bonds (*ca* 1.815 Å; Allen *et al.*, 1987). The C1—N distances reveal a double bonding character. This behavior indicates that the electron density is delocalized over the entire NCS_2_ moiety. The S1—C1—N angle is significantly greater than S2—C1—N probably due to the repulsive interaction between the (–C_6_H_5_)SO_2_ group and the S1 atom, which are in *cis* position in relation to the C1—N bond. Similar behavior is observed in the square-planar platinum(II) and nickel(II) complexes of other dithiocarbimates (Amim *et al.*, 2008; Oliveira *et al.*, 2003, 2004; Franca *et al.*, 2006; Guilardi *et al.*, 2010).

The bond lengths and angles of the tetraphenylphosphonium cations are in agreement with the expected values (Allen *et al.*, 1987).

The crystal packing is made up of discrete oppositely charged units which interact mainly by ionic forces. Moreover, there are two intramolecular (C—H···O) and several intermolecular hydrogen bonding interactions involving the cations, anions and water molecules (C—H···O and C—H···N) (Table 2). Classical hydrogen bonding of the form O—H···O take place between water molecules and anions (Table 2).

The values of bond lengths and angles of the compound under study and its correlative described in the literature with a counter-ion [Bu_4_N]^+^ (Bu is butyl) do not vary significantly (Oliveira *et al.*, 2004). The observed differences are the result of interactions present in the crystal packing of both compounds mainly due to the presence of water molecule of crystallization in the title compound.

### Experimental

The title compound was prepared in water (5 ml) from potassium tetrachloridoplatinate(II) (0.36 mmol), potassium phenylsulfonyldithiocarbimate dihydrate (0.72 mmol) and tetraphenylphosphonium bromide (0.72 mmol). The potassium phenylsulfonyldithiocarbimate dihydrate was prepared from the sulfonamide using procedures described in the literature (Franca *et al.*, 2006). The reaction mixture was stirred for 1 h at room temperature. The yellow solid obtained was filtered, washed with distilled water and dried under reduced pressure. The title compound is soluble in dimethylsulfoxide, slightly soluble in chloroform and insoluble in water and in most organic solvents. Suitable crystals of the title compound were obtained after slow evaporation of a hot chloroform solution. IR spectrum: (most important bands, cm^-1^): 1394 ν(C═N); 1281 ν_ass_(SO_2_); 1142 ν*_sym_*(SO_2_); 935 ν_ass_(CS_2_) and 308 ν(PtS).

### Refinement

All H atoms were fixed geometrically and allowed to ride on their parent atoms, with C—H distances of 0.95Å and O—H distances of 0.85 Å, with *U*_iso_(H) = 1.2 *U*_eq_(C) and *U*_iso_(H) = 1.5 *U*_eq_(O).

### Crystal data

**Table:**

(C_24_H_20_P)_2_[Pt(C_7_H_5_NO_2_S_3_)_2_]·H_2_O	*Z* = 2
*M**_r_* = 1354.45	*F*(000) = 1364
Triclinic, *P*1	*D*_x_ = 1.59 Mg m^−^^3^
Hall symbol: -P 1	Mo *K*α radiation, λ = 0.71073 Å
*a* = 9.2972 (1) Å	Cell parameters from 45459 reflections
*b* = 13.6482 (3) Å	θ = 2.9–26.0°
*c* = 24.4279 (5) Å	µ = 2.81 mm^−^^1^
α = 105.440 (1)°	*T* = 120 K
β = 90.916 (1)°	Prism, yellow
γ = 107.777 (1)°	0.62 × 0.23 × 0.16 mm
*V* = 2829.11 (9) Å^3^	

### Data collection

**Table:**

Nonius KappaCCD diffractometer	9007 reflections with *I* > 2σ(*I*)
ω–scan CCD rotation images, thick slices	*R*_int_ = 0.025
Absorption correction: gaussian (Becker & Coppens, 1974)	θ_max_ = 26.0°, θ_min_ = 3.0°
*T*_min_ = 0.275, *T*_max_ = 0.662	*h* = −11→11
20391 measured reflections	*k* = −16→16
10825 independent reflections	*l* = −29→29

### Refinement

**Table:**

Refinement on *F*^2^	0 restraints
Least-squares matrix: full	H-atom parameters constrained
*R*[*F*^2^ > 2σ(*F*^2^)] = 0.026	*w* = 1/[σ^2^(*F*_o_^2^) + (0.0261*P*)^2^ + 1.8705*P*] where *P* = (*F*_o_^2^ + 2*F*_c_^2^)/3
*wR*(*F*^2^) = 0.063	(Δ/σ)_max_ < 0.001
*S* = 1.03	Δρ_max_ = 0.53 e Å^−^^3^
10825 reflections	Δρ_min_ = −1.41 e Å^−^^3^
706 parameters	

### Special details

**Table:**

Geometry. All e.s.d.'s (except the e.s.d. in the dihedral angle between two l.s. planes) are estimated using the full covariance matrix. The cell e.s.d.'s are taken into account individually in the estimation of e.s.d.'s in distances, angles and torsion angles; correlations between e.s.d.'s in cell parameters are only used when they are defined by crystal symmetry. An approximate (isotropic) treatment of cell e.s.d.'s is used for estimating e.s.d.'s involving l.s. planes.

### Fractional atomic coordinates and isotropic or equivalent isotropic displacement parameters (Å^2^)

**Table:**

	*x*	*y*	*z*	*U*_iso_*/*U*_eq_	
S11	−0.06940 (9)	0.41599 (6)	0.40357 (3)	0.02706 (17)	
S12	0.11144 (9)	0.36578 (6)	0.47859 (3)	0.03094 (18)	
C13	0.1860 (3)	0.4189 (2)	0.28919 (13)	0.0287 (7)	
H13	0.2687	0.4035	0.3042	0.034*	
C11	0.0398 (3)	0.3352 (2)	0.40783 (12)	0.0230 (6)	
C14	0.2089 (4)	0.5150 (3)	0.27555 (14)	0.0384 (8)	
H14	0.3078	0.5659	0.2812	0.046*	
C12	0.0412 (3)	0.3465 (2)	0.28049 (12)	0.0235 (6)	
C17	−0.0809 (3)	0.3676 (2)	0.25841 (13)	0.0300 (7)	
H17	−0.1802	0.3171	0.2528	0.036*	
C15	0.0863 (4)	0.5357 (3)	0.25371 (14)	0.0386 (8)	
H15	0.1017	0.6017	0.2449	0.046*	
S13	0.00917 (8)	0.22849 (6)	0.30179 (3)	0.02565 (16)	
O12	0.1052 (3)	0.17106 (17)	0.27112 (9)	0.0366 (5)	
O11	−0.1508 (2)	0.17218 (17)	0.29224 (10)	0.0385 (6)	
N11	0.0747 (3)	0.26262 (18)	0.36796 (10)	0.0247 (5)	
C16	−0.0564 (4)	0.4629 (3)	0.24468 (14)	0.0362 (8)	
H16	−0.1388	0.4777	0.2289	0.043*	
Pt1	0	0.5	0.5	0.02010 (5)	
Pt2	0.5	0.5	0	0.02475 (5)	
S23	0.81532 (9)	0.71226 (6)	0.20011 (3)	0.03114 (18)	
S22	0.74019 (9)	0.62887 (6)	0.02179 (3)	0.03132 (18)	
S21	0.55254 (9)	0.54490 (6)	0.09806 (3)	0.03022 (18)	
N21	0.8306 (3)	0.70654 (19)	0.13353 (11)	0.0283 (6)	
C24	0.6123 (3)	0.9395 (2)	0.24646 (13)	0.0318 (7)	
H24	0.6381	1.0143	0.2509	0.038*	
O21	0.7619 (3)	0.60907 (18)	0.21057 (10)	0.0414 (6)	
O22	0.9592 (2)	0.78453 (19)	0.23015 (10)	0.0439 (6)	
C23	0.7164 (3)	0.8871 (2)	0.22877 (13)	0.0295 (7)	
H23	0.8144	0.926	0.2215	0.035*	
C21	0.7242 (3)	0.6380 (2)	0.09349 (13)	0.0261 (7)	
C27	0.5358 (4)	0.7204 (2)	0.23234 (13)	0.0318 (7)	
H27	0.5096	0.6454	0.2273	0.038*	
C25	0.4697 (4)	0.8828 (3)	0.25768 (14)	0.0349 (8)	
H25	0.3982	0.9187	0.2702	0.042*	
C22	0.6780 (3)	0.7776 (2)	0.22156 (13)	0.0272 (7)	
C26	0.4324 (4)	0.7733 (3)	0.25046 (14)	0.0349 (8)	
H26	0.3349	0.7343	0.258	0.042*	
C210	−0.2462 (4)	0.1953 (3)	−0.09500 (15)	0.0411 (8)	
H210	−0.1779	0.1939	−0.1235	0.049*	
C226	−0.0696 (3)	0.3418 (2)	0.09037 (12)	0.0242 (6)	
C223	−0.5993 (4)	0.2340 (3)	0.19218 (17)	0.0524 (10)	
H223	−0.6769	0.2361	0.2169	0.063*	
C212	−0.4956 (4)	0.1878 (3)	−0.06802 (16)	0.0481 (10)	
H212	−0.597	0.1835	−0.078	0.058*	
C224	−0.5065 (4)	0.3283 (3)	0.18381 (15)	0.0429 (9)	
H224	−0.5204	0.3947	0.2027	0.051*	
C214	−0.1701 (3)	0.1090 (2)	0.07698 (13)	0.0263 (7)	
C218	−0.1441 (4)	−0.0645 (3)	0.03479 (15)	0.0364 (8)	
H218	−0.1581	−0.125	0.0026	0.044*	
C215	−0.1079 (4)	0.1098 (3)	0.12944 (14)	0.0319 (7)	
H215	−0.0987	0.1684	0.1622	0.038*	
C230	0.1913 (3)	0.4415 (3)	0.12371 (14)	0.0343 (8)	
H230	0.2876	0.4456	0.1396	0.041*	
C229	0.1726 (4)	0.5298 (3)	0.11077 (14)	0.0348 (8)	
H229	0.2558	0.5943	0.1179	0.042*	
C221	−0.4678 (4)	0.1330 (3)	0.12952 (17)	0.0474 (10)	
H221	−0.4545	0.0661	0.1112	0.057*	
C213	−0.4499 (3)	0.1948 (3)	−0.01252 (15)	0.0355 (8)	
H213	−0.5197	0.1941	0.0155	0.043*	
C222	−0.5809 (4)	0.1374 (3)	0.16531 (19)	0.0579 (11)	
H222	−0.6461	0.0733	0.1713	0.069*	
C216	−0.0595 (4)	0.0258 (3)	0.13409 (15)	0.0380 (8)	
H216	−0.0124	0.0284	0.1695	0.046*	
C219	−0.1879 (3)	0.0214 (2)	0.02941 (14)	0.0300 (7)	
H219	−0.2299	0.0203	−0.0065	0.036*	
C211	−0.3956 (4)	0.1871 (3)	−0.10865 (16)	0.0448 (9)	
H211	−0.4291	0.1809	−0.1467	0.054*	
C228	0.0331 (4)	0.5243 (3)	0.08742 (15)	0.0352 (8)	
H228	0.0205	0.5848	0.0783	0.042*	
C225	−0.3934 (3)	0.3257 (3)	0.14790 (14)	0.0335 (7)	
H225	−0.3296	0.3903	0.1419	0.04*	
C227	−0.0881 (3)	0.4307 (2)	0.07732 (14)	0.0307 (7)	
H227	−0.1842	0.427	0.0614	0.037*	
C217	−0.0801 (4)	−0.0623 (3)	0.08676 (16)	0.0390 (8)	
H217	−0.0502	−0.1213	0.0901	0.047*	
C29	−0.1983 (4)	0.2055 (3)	−0.03956 (14)	0.0353 (8)	
H29	−0.0952	0.2143	−0.0293	0.042*	
C28	−0.3011 (3)	0.2029 (2)	0.00162 (13)	0.0268 (7)	
C231	0.0711 (3)	0.3476 (2)	0.11373 (13)	0.0274 (7)	
H231	0.0844	0.2872	0.1228	0.033*	
P2	−0.22894 (8)	0.22070 (6)	0.07315 (3)	0.02458 (17)	
C220	−0.3733 (3)	0.2275 (2)	0.12047 (13)	0.0282 (7)	
P1	0.52139 (8)	0.78466 (6)	0.44818 (3)	0.01989 (16)	
C19	0.6271 (3)	0.7986 (2)	0.55702 (13)	0.0248 (6)	
H19	0.7103	0.7813	0.5397	0.03*	
C123	0.9266 (3)	1.0561 (2)	0.42403 (14)	0.0288 (7)	
H123	1.0122	1.1116	0.4193	0.035*	
C113	0.3828 (3)	0.8245 (2)	0.54829 (13)	0.0247 (6)	
H113	0.3005	0.8265	0.5255	0.03*	
C18	0.5073 (3)	0.8035 (2)	0.52317 (12)	0.0209 (6)	
C114	0.3486 (3)	0.7857 (2)	0.41446 (12)	0.0208 (6)	
C121	0.7655 (3)	0.9723 (2)	0.48515 (12)	0.0226 (6)	
H121	0.7401	0.9707	0.5225	0.027*	
C118	0.2102 (3)	0.8672 (3)	0.36694 (13)	0.0292 (7)	
H118	0.2081	0.9241	0.3517	0.035*	
C125	0.7165 (3)	0.8958 (2)	0.38297 (13)	0.0262 (7)	
H125	0.6564	0.843	0.3503	0.031*	
C127	0.4674 (3)	0.5708 (2)	0.43920 (13)	0.0287 (7)	
H127	0.4043	0.5821	0.4689	0.034*	
C116	0.0808 (3)	0.7009 (3)	0.38610 (14)	0.0334 (7)	
H116	−0.0103	0.644	0.3845	0.04*	
C110	0.6239 (3)	0.8188 (2)	0.61522 (13)	0.0283 (7)	
H110	0.7061	0.8176	0.6383	0.034*	
C119	0.3457 (3)	0.8693 (2)	0.39263 (12)	0.0248 (6)	
H119	0.4359	0.9275	0.3953	0.03*	
C122	0.8882 (3)	1.0545 (2)	0.47799 (13)	0.0269 (7)	
H122	0.9457	1.1097	0.5104	0.032*	
C129	0.5729 (4)	0.4536 (3)	0.37321 (16)	0.0389 (8)	
H129	0.582	0.3844	0.3578	0.047*	
C120	0.6793 (3)	0.8918 (2)	0.43786 (12)	0.0212 (6)	
C124	0.8414 (3)	0.9773 (2)	0.37678 (14)	0.0302 (7)	
H124	0.8687	0.9791	0.3397	0.036*	
C111	0.4991 (4)	0.8411 (2)	0.63990 (13)	0.0308 (7)	
H111	0.4966	0.8554	0.6801	0.037*	
C112	0.3796 (3)	0.8426 (2)	0.60664 (13)	0.0290 (7)	
H112	0.2941	0.8561	0.6239	0.035*	
C130	0.6522 (4)	0.5373 (3)	0.35300 (16)	0.0418 (9)	
H130	0.7154	0.5255	0.3234	0.05*	
C115	0.2154 (3)	0.7011 (2)	0.41123 (13)	0.0288 (7)	
H115	0.2172	0.644	0.4262	0.035*	
C117	0.0783 (3)	0.7833 (3)	0.36323 (13)	0.0322 (7)	
H117	−0.0138	0.7819	0.3451	0.039*	
C131	0.6405 (3)	0.6386 (2)	0.37554 (15)	0.0348 (8)	
H131	0.6955	0.6962	0.3614	0.042*	
C126	0.5483 (3)	0.6559 (2)	0.41880 (12)	0.0234 (6)	
C128	0.4798 (4)	0.4700 (2)	0.41595 (15)	0.0353 (8)	
H128	0.424	0.4118	0.4294	0.042*	
OW	0.1927 (3)	0.9828 (2)	0.24598 (14)	0.0654 (8)	
H1W	0.1616	1.0362	0.2587	0.098*	
H2W	0.1166	0.9262	0.2403	0.098*	

### Atomic displacement parameters (Å^2^)

**Table:**

	*U*^11^	*U*^22^	*U*^33^	*U*^12^	*U*^13^	*U*^23^
S11	0.0321 (4)	0.0306 (4)	0.0206 (4)	0.0183 (3)	−0.0002 (3)	0.0015 (3)
S12	0.0449 (5)	0.0327 (4)	0.0234 (4)	0.0256 (4)	0.0034 (3)	0.0062 (3)
C13	0.0295 (17)	0.0304 (17)	0.0269 (17)	0.0086 (13)	0.0040 (13)	0.0103 (14)
C11	0.0257 (15)	0.0201 (15)	0.0240 (16)	0.0067 (12)	0.0061 (12)	0.0083 (12)
C14	0.043 (2)	0.0314 (18)	0.035 (2)	0.0007 (15)	0.0058 (16)	0.0134 (15)
C12	0.0289 (16)	0.0242 (16)	0.0186 (16)	0.0107 (13)	0.0060 (12)	0.0053 (12)
C17	0.0314 (17)	0.0303 (17)	0.0265 (17)	0.0121 (14)	0.0044 (13)	0.0024 (14)
C15	0.060 (2)	0.0267 (18)	0.0317 (19)	0.0149 (16)	0.0049 (16)	0.0115 (15)
S13	0.0331 (4)	0.0189 (4)	0.0239 (4)	0.0072 (3)	0.0073 (3)	0.0055 (3)
O12	0.0575 (15)	0.0304 (12)	0.0294 (13)	0.0245 (11)	0.0175 (11)	0.0081 (10)
O11	0.0365 (13)	0.0307 (12)	0.0357 (14)	−0.0053 (10)	0.0027 (10)	0.0076 (10)
N11	0.0356 (14)	0.0202 (13)	0.0230 (14)	0.0133 (11)	0.0086 (11)	0.0083 (11)
C16	0.047 (2)	0.0385 (19)	0.0269 (18)	0.0214 (16)	0.0003 (15)	0.0074 (15)
Pt1	0.02270 (9)	0.01875 (8)	0.02005 (9)	0.00908 (6)	0.00353 (6)	0.00460 (6)
Pt2	0.02283 (9)	0.02280 (9)	0.02672 (10)	0.00679 (7)	0.00329 (7)	0.00452 (7)
S23	0.0321 (4)	0.0295 (4)	0.0315 (5)	0.0098 (3)	−0.0047 (3)	0.0087 (3)
S22	0.0271 (4)	0.0309 (4)	0.0303 (4)	0.0032 (3)	0.0060 (3)	0.0065 (3)
S21	0.0274 (4)	0.0310 (4)	0.0282 (4)	0.0040 (3)	0.0033 (3)	0.0080 (3)
N21	0.0243 (13)	0.0267 (14)	0.0327 (15)	0.0083 (11)	0.0017 (11)	0.0065 (12)
C24	0.0392 (19)	0.0214 (16)	0.0281 (18)	0.0044 (14)	−0.0017 (14)	0.0025 (13)
O21	0.0512 (14)	0.0353 (13)	0.0449 (15)	0.0173 (11)	0.0005 (11)	0.0196 (11)
O22	0.0341 (13)	0.0461 (15)	0.0435 (15)	0.0053 (11)	−0.0129 (11)	0.0093 (12)
C23	0.0308 (17)	0.0260 (16)	0.0259 (17)	0.0008 (13)	−0.0005 (13)	0.0075 (13)
C21	0.0276 (16)	0.0253 (16)	0.0305 (18)	0.0160 (13)	0.0046 (13)	0.0074 (13)
C27	0.0406 (19)	0.0233 (16)	0.0256 (18)	0.0028 (14)	0.0020 (14)	0.0059 (14)
C25	0.042 (2)	0.0318 (18)	0.0280 (18)	0.0122 (15)	0.0036 (14)	0.0032 (14)
C22	0.0346 (17)	0.0225 (16)	0.0220 (16)	0.0061 (13)	−0.0022 (13)	0.0060 (13)
C26	0.0354 (19)	0.0322 (18)	0.0323 (19)	0.0028 (14)	0.0102 (14)	0.0099 (15)
C210	0.054 (2)	0.043 (2)	0.031 (2)	0.0189 (17)	0.0100 (16)	0.0155 (16)
C226	0.0238 (16)	0.0275 (16)	0.0226 (16)	0.0099 (12)	0.0068 (12)	0.0072 (13)
C223	0.045 (2)	0.063 (3)	0.057 (3)	0.023 (2)	0.0304 (19)	0.023 (2)
C212	0.037 (2)	0.054 (2)	0.053 (3)	0.0080 (17)	−0.0110 (18)	0.023 (2)
C224	0.042 (2)	0.050 (2)	0.046 (2)	0.0256 (18)	0.0173 (17)	0.0158 (18)
C214	0.0250 (16)	0.0267 (16)	0.0297 (18)	0.0079 (12)	0.0066 (13)	0.0123 (14)
C218	0.0398 (19)	0.0285 (17)	0.041 (2)	0.0125 (15)	0.0131 (15)	0.0078 (15)
C215	0.0382 (18)	0.0301 (17)	0.0277 (18)	0.0098 (14)	0.0037 (14)	0.0100 (14)
C230	0.0252 (17)	0.043 (2)	0.037 (2)	0.0088 (14)	0.0053 (14)	0.0162 (16)
C229	0.0288 (18)	0.0300 (18)	0.041 (2)	0.0052 (14)	0.0094 (14)	0.0073 (15)
C221	0.049 (2)	0.033 (2)	0.059 (3)	0.0096 (16)	0.0254 (19)	0.0147 (18)
C213	0.0267 (17)	0.0395 (19)	0.040 (2)	0.0072 (14)	0.0009 (14)	0.0151 (16)
C222	0.052 (2)	0.045 (2)	0.077 (3)	0.0085 (19)	0.038 (2)	0.023 (2)
C216	0.0354 (19)	0.042 (2)	0.044 (2)	0.0117 (15)	0.0034 (15)	0.0259 (18)
C219	0.0304 (17)	0.0313 (17)	0.0295 (18)	0.0100 (14)	0.0058 (13)	0.0101 (14)
C211	0.052 (2)	0.044 (2)	0.034 (2)	0.0053 (17)	−0.0069 (17)	0.0168 (17)
C228	0.0340 (19)	0.0284 (17)	0.048 (2)	0.0139 (14)	0.0114 (15)	0.0140 (16)
C225	0.0315 (18)	0.0385 (19)	0.037 (2)	0.0165 (15)	0.0076 (14)	0.0149 (16)
C227	0.0243 (16)	0.0318 (18)	0.040 (2)	0.0117 (13)	0.0083 (14)	0.0139 (15)
C217	0.0386 (19)	0.037 (2)	0.055 (2)	0.0194 (15)	0.0149 (17)	0.0261 (18)
C29	0.0371 (19)	0.041 (2)	0.0307 (19)	0.0160 (15)	0.0038 (14)	0.0112 (15)
C28	0.0278 (16)	0.0237 (16)	0.0294 (17)	0.0072 (12)	0.0016 (13)	0.0096 (13)
C231	0.0282 (17)	0.0321 (17)	0.0260 (17)	0.0122 (13)	0.0059 (13)	0.0120 (14)
P2	0.0243 (4)	0.0257 (4)	0.0249 (4)	0.0090 (3)	0.0046 (3)	0.0078 (3)
C220	0.0257 (16)	0.0327 (17)	0.0282 (17)	0.0105 (13)	0.0063 (13)	0.0104 (14)
P1	0.0186 (4)	0.0179 (4)	0.0238 (4)	0.0060 (3)	0.0019 (3)	0.0070 (3)
C19	0.0228 (15)	0.0202 (15)	0.0320 (18)	0.0076 (12)	0.0023 (12)	0.0078 (13)
C123	0.0225 (16)	0.0243 (16)	0.041 (2)	0.0058 (12)	0.0000 (13)	0.0145 (14)
C113	0.0229 (15)	0.0261 (16)	0.0297 (17)	0.0095 (12)	0.0043 (12)	0.0135 (13)
C18	0.0219 (15)	0.0165 (14)	0.0244 (16)	0.0068 (11)	0.0026 (11)	0.0052 (12)
C114	0.0199 (14)	0.0224 (15)	0.0202 (15)	0.0092 (11)	0.0012 (11)	0.0037 (12)
C121	0.0243 (15)	0.0213 (15)	0.0234 (16)	0.0084 (12)	0.0010 (12)	0.0072 (12)
C118	0.0330 (18)	0.0355 (18)	0.0281 (18)	0.0173 (14)	0.0050 (13)	0.0165 (14)
C125	0.0258 (16)	0.0272 (16)	0.0249 (17)	0.0074 (13)	0.0001 (12)	0.0078 (13)
C127	0.0330 (17)	0.0258 (16)	0.0282 (18)	0.0105 (13)	0.0017 (13)	0.0082 (14)
C116	0.0220 (16)	0.0328 (18)	0.040 (2)	0.0036 (13)	−0.0026 (13)	0.0074 (15)
C110	0.0341 (17)	0.0229 (16)	0.0284 (18)	0.0102 (13)	−0.0051 (13)	0.0072 (13)
C119	0.0258 (16)	0.0246 (15)	0.0254 (17)	0.0094 (12)	0.0053 (12)	0.0079 (13)
C122	0.0254 (16)	0.0199 (15)	0.0351 (19)	0.0068 (12)	−0.0036 (13)	0.0087 (13)
C129	0.0340 (19)	0.0239 (17)	0.055 (2)	0.0138 (14)	−0.0060 (16)	0.0003 (16)
C120	0.0186 (14)	0.0203 (15)	0.0272 (17)	0.0081 (11)	0.0031 (12)	0.0086 (12)
C124	0.0273 (16)	0.0344 (18)	0.0334 (19)	0.0097 (14)	0.0059 (13)	0.0174 (15)
C111	0.047 (2)	0.0239 (16)	0.0237 (17)	0.0139 (14)	0.0058 (14)	0.0074 (13)
C112	0.0341 (17)	0.0274 (17)	0.0313 (18)	0.0146 (13)	0.0104 (14)	0.0122 (14)
C130	0.0305 (18)	0.0304 (19)	0.059 (2)	0.0108 (15)	0.0156 (16)	0.0020 (17)
C115	0.0248 (16)	0.0244 (16)	0.0355 (19)	0.0040 (12)	−0.0011 (13)	0.0106 (14)
C117	0.0277 (17)	0.044 (2)	0.0272 (18)	0.0173 (15)	−0.0040 (13)	0.0076 (15)
C131	0.0270 (17)	0.0262 (17)	0.048 (2)	0.0075 (13)	0.0122 (15)	0.0062 (15)
C126	0.0203 (15)	0.0207 (15)	0.0271 (17)	0.0067 (12)	−0.0022 (12)	0.0033 (12)
C128	0.043 (2)	0.0222 (17)	0.040 (2)	0.0086 (14)	−0.0025 (15)	0.0102 (15)
OW	0.0456 (15)	0.0403 (15)	0.120 (3)	0.0208 (12)	0.0086 (15)	0.0302 (16)

### Geometric parameters (Å, °)

**Table:**

S11—C11	1.732 (3)	C221—C222	1.383 (5)
S11—Pt1	2.3111 (7)	C221—C220	1.397 (4)
S12—C11	1.736 (3)	C221—H221	0.95
S12—Pt1	2.3171 (7)	C213—C28	1.385 (4)
C13—C12	1.378 (4)	C213—H213	0.95
C13—C14	1.394 (4)	C222—H222	0.95
C13—H13	0.95	C216—C217	1.390 (5)
C11—N11	1.314 (4)	C216—H216	0.95
C14—C15	1.386 (5)	C219—H219	0.95
C14—H14	0.95	C211—H211	0.95
C12—C17	1.387 (4)	C228—C227	1.380 (4)
C12—S13	1.764 (3)	C228—H228	0.95
C17—C16	1.382 (4)	C225—C220	1.399 (4)
C17—H17	0.95	C225—H225	0.95
C15—C16	1.365 (5)	C227—H227	0.95
C15—H15	0.95	C217—H217	0.95
S13—O11	1.433 (2)	C29—C28	1.399 (4)
S13—O12	1.452 (2)	C29—H29	0.95
S13—N11	1.611 (3)	C28—P2	1.789 (3)
C16—H16	0.95	C231—H231	0.95
Pt1—S11^i^	2.3111 (7)	P2—C220	1.793 (3)
Pt1—S12^i^	2.3171 (7)	P1—C18	1.793 (3)
Pt2—S21^ii^	2.3130 (8)	P1—C114	1.800 (3)
Pt2—S21	2.3130 (8)	P1—C120	1.804 (3)
Pt2—S22^ii^	2.3299 (7)	P1—C126	1.808 (3)
Pt2—S22	2.3299 (7)	C19—C110	1.377 (4)
S23—O21	1.437 (2)	C19—C18	1.404 (4)
S23—O22	1.442 (2)	C19—H19	0.95
S23—N21	1.618 (3)	C123—C122	1.375 (4)
S23—C22	1.780 (3)	C123—C124	1.381 (4)
S22—C21	1.735 (3)	C123—H123	0.95
S21—C21	1.737 (3)	C113—C112	1.383 (4)
N21—C21	1.314 (4)	C113—C18	1.390 (4)
C24—C23	1.382 (4)	C113—H113	0.95
C24—C25	1.389 (4)	C114—C119	1.388 (4)
C24—H24	0.95	C114—C115	1.396 (4)
C23—C22	1.387 (4)	C121—C122	1.385 (4)
C23—H23	0.95	C121—C120	1.393 (4)
C27—C26	1.382 (4)	C121—H121	0.95
C27—C22	1.384 (4)	C118—C117	1.382 (4)
C27—H27	0.95	C118—C119	1.387 (4)
C25—C26	1.388 (4)	C118—H118	0.95
C25—H25	0.95	C125—C124	1.384 (4)
C26—H26	0.95	C125—C120	1.401 (4)
C210—C29	1.376 (5)	C125—H125	0.95
C210—C211	1.387 (5)	C127—C128	1.382 (4)
C210—H210	0.95	C127—C126	1.396 (4)
C226—C231	1.390 (4)	C127—H127	0.95
C226—C227	1.391 (4)	C116—C115	1.384 (4)
C226—P2	1.794 (3)	C116—C117	1.388 (4)
C223—C222	1.370 (5)	C116—H116	0.95
C223—C224	1.382 (5)	C110—C111	1.394 (4)
C223—H223	0.95	C110—H110	0.95
C212—C211	1.371 (5)	C119—H119	0.95
C212—C213	1.384 (5)	C122—H122	0.95
C212—H212	0.95	C129—C130	1.375 (5)
C224—C225	1.383 (4)	C129—C128	1.383 (5)
C224—H224	0.95	C129—H129	0.95
C214—C219	1.393 (4)	C124—H124	0.95
C214—C215	1.394 (4)	C111—C112	1.374 (4)
C214—P2	1.793 (3)	C111—H111	0.95
C218—C217	1.383 (5)	C112—H112	0.95
C218—C219	1.388 (4)	C130—C131	1.384 (4)
C218—H218	0.95	C130—H130	0.95
C215—C216	1.382 (4)	C115—H115	0.95
C215—H215	0.95	C117—H117	0.95
C230—C231	1.378 (4)	C131—C126	1.388 (4)
C230—C229	1.382 (4)	C131—H131	0.95
C230—H230	0.95	C128—H128	0.95
C229—C228	1.381 (4)	OW—H1W	0.85
C229—H229	0.95	OW—H2W	0.85
			
C11—S11—Pt1	88.30 (10)	C218—C219—H219	120.1
C11—S12—Pt1	88.00 (10)	C214—C219—H219	120.1
C12—C13—C14	118.9 (3)	C212—C211—C210	121.0 (3)
C12—C13—H13	120.6	C212—C211—H211	119.5
C14—C13—H13	120.6	C210—C211—H211	119.5
N11—C11—S11	131.0 (2)	C227—C228—C229	120.0 (3)
N11—C11—S12	120.4 (2)	C227—C228—H228	120
S11—C11—S12	108.57 (16)	C229—C228—H228	120
C15—C14—C13	119.5 (3)	C224—C225—C220	119.6 (3)
C15—C14—H14	120.2	C224—C225—H225	120.2
C13—C14—H14	120.2	C220—C225—H225	120.2
C13—C12—C17	121.3 (3)	C228—C227—C226	120.1 (3)
C13—C12—S13	119.1 (2)	C228—C227—H227	120
C17—C12—S13	119.4 (2)	C226—C227—H227	120
C16—C17—C12	119.2 (3)	C218—C217—C216	120.2 (3)
C16—C17—H17	120.4	C218—C217—H217	119.9
C12—C17—H17	120.4	C216—C217—H217	119.9
C16—C15—C14	121.1 (3)	C210—C29—C28	120.1 (3)
C16—C15—H15	119.5	C210—C29—H29	119.9
C14—C15—H15	119.5	C28—C29—H29	119.9
O11—S13—O12	116.04 (14)	C213—C28—C29	120.2 (3)
O11—S13—N11	112.57 (13)	C213—C28—P2	122.1 (2)
O12—S13—N11	104.85 (13)	C29—C28—P2	117.4 (2)
O11—S13—C12	107.86 (14)	C230—C231—C226	119.7 (3)
O12—S13—C12	107.06 (13)	C230—C231—H231	120.2
N11—S13—C12	108.09 (13)	C226—C231—H231	120.2
C11—N11—S13	123.0 (2)	C28—P2—C214	110.42 (14)
C15—C16—C17	120.1 (3)	C28—P2—C220	110.60 (14)
C15—C16—H16	120	C214—P2—C220	107.18 (14)
C17—C16—H16	120	C28—P2—C226	105.17 (14)
S11^i^—Pt1—S11	180	C214—P2—C226	110.64 (14)
S11^i^—Pt1—S12^i^	74.95 (3)	C220—P2—C226	112.87 (14)
S11—Pt1—S12^i^	105.05 (3)	C221—C220—C225	119.8 (3)
S11^i^—Pt1—S12	105.05 (3)	C221—C220—P2	119.2 (2)
S11—Pt1—S12	74.95 (3)	C225—C220—P2	121.0 (2)
S12^i^—Pt1—S12	180	C18—P1—C114	109.44 (13)
S21^ii^—Pt2—S21	180.00 (4)	C18—P1—C120	109.39 (13)
S21^ii^—Pt2—S22^ii^	74.22 (3)	C114—P1—C120	109.50 (13)
S21—Pt2—S22^ii^	105.78 (3)	C18—P1—C126	107.80 (13)
S21^ii^—Pt2—S22	105.78 (3)	C114—P1—C126	109.72 (13)
S21—Pt2—S22	74.22 (3)	C120—P1—C126	110.96 (13)
S22^ii^—Pt2—S22	180.00 (4)	C110—C19—C18	120.1 (3)
O21—S23—O22	116.76 (14)	C110—C19—H19	119.9
O21—S23—N21	113.99 (14)	C18—C19—H19	119.9
O22—S23—N21	105.31 (14)	C122—C123—C124	120.2 (3)
O21—S23—C22	107.20 (14)	C122—C123—H123	119.9
O22—S23—C22	106.57 (14)	C124—C123—H123	119.9
N21—S23—C22	106.34 (13)	C112—C113—C18	119.5 (3)
C21—S22—Pt2	88.79 (10)	C112—C113—H113	120.2
C21—S21—Pt2	89.30 (11)	C18—C113—H113	120.2
C21—N21—S23	120.9 (2)	C113—C18—C19	119.8 (3)
C23—C24—C25	120.0 (3)	C113—C18—P1	121.6 (2)
C23—C24—H24	120	C19—C18—P1	118.6 (2)
C25—C24—H24	120	C119—C114—C115	120.1 (3)
C24—C23—C22	120.0 (3)	C119—C114—P1	121.3 (2)
C24—C23—H23	120	C115—C114—P1	118.7 (2)
C22—C23—H23	120	C122—C121—C120	120.3 (3)
N21—C21—S22	121.6 (2)	C122—C121—H121	119.8
N21—C21—S21	130.8 (2)	C120—C121—H121	119.8
S22—C21—S21	107.60 (17)	C117—C118—C119	120.6 (3)
C26—C27—C22	119.5 (3)	C117—C118—H118	119.7
C26—C27—H27	120.3	C119—C118—H118	119.7
C22—C27—H27	120.3	C124—C125—C120	119.4 (3)
C26—C25—C24	119.6 (3)	C124—C125—H125	120.3
C26—C25—H25	120.2	C120—C125—H125	120.3
C24—C25—H25	120.2	C128—C127—C126	119.5 (3)
C27—C22—C23	120.4 (3)	C128—C127—H127	120.2
C27—C22—S23	120.4 (2)	C126—C127—H127	120.2
C23—C22—S23	119.2 (2)	C115—C116—C117	120.3 (3)
C27—C26—C25	120.6 (3)	C115—C116—H116	119.8
C27—C26—H26	119.7	C117—C116—H116	119.8
C25—C26—H26	119.7	C19—C110—C111	119.4 (3)
C29—C210—C211	119.0 (3)	C19—C110—H110	120.3
C29—C210—H210	120.5	C111—C110—H110	120.3
C211—C210—H210	120.5	C118—C119—C114	119.6 (3)
C231—C226—C227	119.8 (3)	C118—C119—H119	120.2
C231—C226—P2	122.2 (2)	C114—C119—H119	120.2
C227—C226—P2	117.9 (2)	C123—C122—C121	120.0 (3)
C222—C223—C224	120.8 (3)	C123—C122—H122	120
C222—C223—H223	119.6	C121—C122—H122	120
C224—C223—H223	119.6	C130—C129—C128	120.1 (3)
C211—C212—C213	120.5 (3)	C130—C129—H129	120
C211—C212—H212	119.8	C128—C129—H129	120
C213—C212—H212	119.8	C121—C120—C125	119.3 (3)
C223—C224—C225	119.9 (3)	C121—C120—P1	119.6 (2)
C223—C224—H224	120	C125—C120—P1	121.1 (2)
C225—C224—H224	120	C123—C124—C125	120.6 (3)
C219—C214—C215	119.6 (3)	C123—C124—H124	119.7
C219—C214—P2	121.8 (2)	C125—C124—H124	119.7
C215—C214—P2	118.6 (2)	C112—C111—C110	120.6 (3)
C217—C218—C219	120.3 (3)	C112—C111—H111	119.7
C217—C218—H218	119.8	C110—C111—H111	119.7
C219—C218—H218	119.8	C111—C112—C113	120.6 (3)
C216—C215—C214	120.4 (3)	C111—C112—H112	119.7
C216—C215—H215	119.8	C113—C112—H112	119.7
C214—C215—H215	119.8	C129—C130—C131	120.3 (3)
C231—C230—C229	120.5 (3)	C129—C130—H130	119.8
C231—C230—H230	119.8	C131—C130—H130	119.8
C229—C230—H230	119.8	C116—C115—C114	119.7 (3)
C228—C229—C230	120.1 (3)	C116—C115—H115	120.2
C228—C229—H229	120	C114—C115—H115	120.2
C230—C229—H229	120	C118—C117—C116	119.7 (3)
C222—C221—C220	119.6 (3)	C118—C117—H117	120.1
C222—C221—H221	120.2	C116—C117—H117	120.1
C220—C221—H221	120.2	C130—C131—C126	119.8 (3)
C212—C213—C28	119.1 (3)	C130—C131—H131	120.1
C212—C213—H213	120.5	C126—C131—H131	120.1
C28—C213—H213	120.5	C131—C126—C127	119.8 (3)
C223—C222—C221	120.3 (3)	C131—C126—P1	122.3 (2)
C223—C222—H222	119.8	C127—C126—P1	117.9 (2)
C221—C222—H222	119.8	C127—C128—C129	120.4 (3)
C215—C216—C217	119.7 (3)	C127—C128—H128	119.8
C215—C216—H216	120.1	C129—C128—H128	119.8
C217—C216—H216	120.1	H1W—OW—H2W	107.7
C218—C219—C214	119.7 (3)		
			
Pt1—S11—C11—N11	−174.7 (3)	C29—C28—P2—C214	−67.0 (3)
Pt1—S11—C11—S12	3.68 (13)	C213—C28—P2—C220	−0.7 (3)
Pt1—S12—C11—N11	174.9 (2)	C29—C28—P2—C220	174.5 (2)
Pt1—S12—C11—S11	−3.67 (13)	C213—C28—P2—C226	−122.9 (3)
C12—C13—C14—C15	0.1 (5)	C29—C28—P2—C226	52.4 (3)
C14—C13—C12—C17	0.2 (4)	C219—C214—P2—C28	−2.5 (3)
C14—C13—C12—S13	−176.1 (2)	C215—C214—P2—C28	178.8 (2)
C13—C12—C17—C16	0.3 (4)	C219—C214—P2—C220	118.1 (3)
S13—C12—C17—C16	176.5 (2)	C215—C214—P2—C220	−60.6 (3)
C13—C14—C15—C16	−0.8 (5)	C219—C214—P2—C226	−118.5 (2)
C13—C12—S13—O11	175.5 (2)	C215—C214—P2—C226	62.8 (3)
C17—C12—S13—O11	−0.8 (3)	C231—C226—P2—C28	−130.5 (2)
C13—C12—S13—O12	−58.9 (3)	C227—C226—P2—C28	46.7 (3)
C17—C12—S13—O12	124.7 (2)	C231—C226—P2—C214	−11.2 (3)
C13—C12—S13—N11	53.5 (3)	C227—C226—P2—C214	166.0 (2)
C17—C12—S13—N11	−122.8 (2)	C231—C226—P2—C220	108.9 (3)
S11—C11—N11—S13	−3.3 (4)	C227—C226—P2—C220	−73.9 (3)
S12—C11—N11—S13	178.56 (15)	C222—C221—C220—C225	0.4 (6)
O11—S13—N11—C11	−68.1 (3)	C222—C221—C220—P2	−179.1 (3)
O12—S13—N11—C11	164.8 (2)	C224—C225—C220—C221	0.1 (5)
C12—S13—N11—C11	50.9 (3)	C224—C225—C220—P2	179.5 (3)
C14—C15—C16—C17	1.3 (5)	C28—P2—C220—C221	89.8 (3)
C12—C17—C16—C15	−1.0 (5)	C214—P2—C220—C221	−30.6 (3)
C11—S11—Pt1—S12^i^	177.30 (9)	C226—P2—C220—C221	−152.7 (3)
C11—S11—Pt1—S12	−2.70 (9)	C28—P2—C220—C225	−89.6 (3)
C11—S12—Pt1—S11^i^	−177.30 (9)	C214—P2—C220—C225	149.9 (3)
C11—S12—Pt1—S11	2.70 (9)	C226—P2—C220—C225	27.9 (3)
S21^ii^—Pt2—S22—C21	178.15 (10)	C112—C113—C18—C19	1.3 (4)
S21—Pt2—S22—C21	−1.85 (10)	C112—C113—C18—P1	−177.5 (2)
S22^ii^—Pt2—S21—C21	−178.15 (9)	C110—C19—C18—C113	−2.6 (4)
S22—Pt2—S21—C21	1.85 (9)	C110—C19—C18—P1	176.3 (2)
O21—S23—N21—C21	43.2 (3)	C114—P1—C18—C113	−5.1 (3)
O22—S23—N21—C21	172.5 (2)	C120—P1—C18—C113	114.9 (2)
C22—S23—N21—C21	−74.7 (3)	C126—P1—C18—C113	−124.3 (2)
C25—C24—C23—C22	0.8 (5)	C114—P1—C18—C19	176.1 (2)
S23—N21—C21—S22	−178.76 (15)	C120—P1—C18—C19	−63.9 (2)
S23—N21—C21—S21	1.4 (4)	C126—P1—C18—C19	56.9 (2)
Pt2—S22—C21—N21	−177.4 (2)	C18—P1—C114—C119	111.1 (2)
Pt2—S22—C21—S21	2.49 (13)	C120—P1—C114—C119	−8.8 (3)
Pt2—S21—C21—N21	177.4 (3)	C126—P1—C114—C119	−130.8 (2)
Pt2—S21—C21—S22	−2.51 (13)	C18—P1—C114—C115	−68.0 (3)
C23—C24—C25—C26	−0.7 (5)	C120—P1—C114—C115	172.1 (2)
C26—C27—C22—C23	−0.1 (5)	C126—P1—C114—C115	50.1 (3)
C26—C27—C22—S23	178.2 (2)	C18—C19—C110—C111	1.8 (4)
C24—C23—C22—C27	−0.4 (5)	C117—C118—C119—C114	0.4 (5)
C24—C23—C22—S23	−178.8 (2)	C115—C114—C119—C118	−0.9 (4)
O21—S23—C22—C27	−13.4 (3)	P1—C114—C119—C118	180.0 (2)
O22—S23—C22—C27	−139.1 (3)	C124—C123—C122—C121	1.5 (4)
N21—S23—C22—C27	108.9 (3)	C120—C121—C122—C123	−0.9 (4)
O21—S23—C22—C23	165.0 (2)	C122—C121—C120—C125	−1.0 (4)
O22—S23—C22—C23	39.3 (3)	C122—C121—C120—P1	179.3 (2)
N21—S23—C22—C23	−72.7 (3)	C124—C125—C120—C121	2.3 (4)
C22—C27—C26—C25	0.2 (5)	C124—C125—C120—P1	−177.9 (2)
C24—C25—C26—C27	0.1 (5)	C18—P1—C120—C121	−0.8 (3)
C222—C223—C224—C225	0.0 (6)	C114—P1—C120—C121	119.1 (2)
C219—C214—C215—C216	2.0 (5)	C126—P1—C120—C121	−119.7 (2)
P2—C214—C215—C216	−179.2 (2)	C18—P1—C120—C125	179.4 (2)
C231—C230—C229—C228	−0.3 (5)	C114—P1—C120—C125	−60.7 (3)
C211—C212—C213—C28	1.1 (5)	C126—P1—C120—C125	60.6 (3)
C224—C223—C222—C221	0.4 (7)	C122—C123—C124—C125	0.0 (4)
C220—C221—C222—C223	−0.6 (7)	C120—C125—C124—C123	−1.9 (4)
C214—C215—C216—C217	−3.2 (5)	C19—C110—C111—C112	0.3 (4)
C217—C218—C219—C214	−1.3 (5)	C110—C111—C112—C113	−1.6 (4)
C215—C214—C219—C218	0.3 (4)	C18—C113—C112—C111	0.8 (4)
P2—C214—C219—C218	−178.4 (2)	C128—C129—C130—C131	0.5 (5)
C213—C212—C211—C210	−1.2 (6)	C117—C116—C115—C114	1.2 (5)
C29—C210—C211—C212	−0.7 (5)	C119—C114—C115—C116	0.1 (4)
C230—C229—C228—C227	0.4 (5)	P1—C114—C115—C116	179.2 (2)
C223—C224—C225—C220	−0.3 (5)	C119—C118—C117—C116	0.9 (5)
C229—C228—C227—C226	−0.3 (5)	C115—C116—C117—C118	−1.7 (5)
C231—C226—C227—C228	0.2 (5)	C129—C130—C131—C126	0.0 (5)
P2—C226—C227—C228	−177.1 (2)	C130—C131—C126—C127	−0.2 (5)
C219—C218—C217—C216	0.1 (5)	C130—C131—C126—P1	−177.4 (3)
C215—C216—C217—C218	2.2 (5)	C128—C127—C126—C131	−0.2 (4)
C211—C210—C29—C28	2.7 (5)	C128—C127—C126—P1	177.1 (2)
C212—C213—C28—C29	0.9 (5)	C18—P1—C126—C131	−143.9 (3)
C212—C213—C28—P2	176.0 (3)	C114—P1—C126—C131	97.0 (3)
C210—C29—C28—C213	−2.8 (5)	C120—P1—C126—C131	−24.1 (3)
C210—C29—C28—P2	−178.2 (3)	C18—P1—C126—C127	38.9 (3)
C229—C230—C231—C226	0.1 (5)	C114—P1—C126—C127	−80.2 (3)
C227—C226—C231—C230	−0.1 (4)	C120—P1—C126—C127	158.7 (2)
P2—C226—C231—C230	177.1 (2)	C126—C127—C128—C129	0.7 (5)
C213—C28—P2—C214	117.8 (3)	C130—C129—C128—C127	−0.9 (5)

Symmetry codes: (i) −*x*, −*y*+1, −*z*+1; (ii) −*x*+1, −*y*+1, −*z*.

### Hydrogen-bond geometry (Å, °)

**Table:**

*D*—H···*A*	*D*—H	H···*A*	*D*···*A*	*D*—H···*A*
OW—H1W···O12^iii^	0.85	2.01	2.845 (3)	166
OW—H2W···O22^iv^	0.85	1.99	2.831 (4)	172
C16—H16···O21^iv^	0.95	2.39	3.230 (4)	148
C17—H17···O11	0.95	2.5	2.889 (4)	105
C24—H24···O11^v^	0.95	2.39	3.154 (4)	137
C25—H25···OW	0.95	2.46	3.303 (4)	149
C27—H27···O21	0.95	2.56	2.926 (4)	103
C110—H110···N11^vi^	0.95	2.58	3.371 (4)	141
C112—H112···O11^i^	0.95	2.51	3.292 (4)	139
C123—H123···N11^v^	0.95	2.61	3.385 (4)	139

Symmetry codes: (iii) *x*, *y*+1, *z*; (iv) *x*−1, *y*, *z*; (v) *x*+1, *y*+1, *z*; (vi) −*x*+1, −*y*+1, −*z*+1; (i) −*x*, −*y*+1, −*z*+1.
